# Identification and Genetic Characterization of a Strain of African Horse Sickness Virus Serotype 1 and Its Safety Evaluation in a Mouse Model

**DOI:** 10.3390/microorganisms13102314

**Published:** 2025-10-06

**Authors:** Min Zhang, Xue-Feng Wang, Si-Fan Guo, Lei Wang, Bo-Fan Fu, Jing-Wen Wang, Ya-Fen Song, Xiao-Yue Yang, Si-Yuan Hao, Qian-Yi Zhang, Bing Zhang, Cheng-Huai Yang

**Affiliations:** 1 National Center for Veterinary Culture Collection, China Institute of Veterinary Drug Control, Beijing 102629, China; zmbooksea@sina.com (M.Z.); 89gsf2008@sina.com (S.-F.G.); sdsfdxwl@163.com (L.W.); fubofan2025@163.com (B.-F.F.); jw_wang_cava@yeah.net (J.-W.W.); songyafen1@126.com (Y.-F.S.); xiaoyue_yang2025@126.com (X.-Y.Y.); 18201098620@163.com (S.-Y.H.); zhangqy114@126.com (Q.-Y.Z.); 2 State Key Laboratory for Animal Disease Control and Prevention, Harbin Veterinary Research Institute, The Chinese Academy of Agricultural Sciences, Harbin 150069, China; wangxuefeng@caas.cn

**Keywords:** African horse sickness virus, identification, genetic characterization, safety evaluation

## Abstract

African horse sickness (AHS) is an arthropod-borne, severe equid disease caused by African horse sickness virus (AHSV). AHSV has high mortality and is endemic to sub-Saharan Africa. It has been classified into nine distinct serotypes (AHSV-1 to AHSV-9) based on VP2 immunogenicity. The AHS outbreak in Thailand in 2020, caused by AHSV-1, marked the first occurrence of this disease in Southeast Asia. It posed a substantial threat to the security of the equine industry in the nations across the region. To ensure the emergency reserve for AHS prevention and control, the AHSV strain imported to China from abroad over 60 years ago was characterized in this study. The strain was passaged in mice and then blind-passaged in Vero cells. The plaque purification method was then used to purify the strain and obtain its cell-adapted version, named AHSV/C. Neutralization tests confirmed that the virus belongs to AHSV-1. Whole-genome sequencing revealed that AHSV/C was highly homologous to AHSV-1 isolate 1180, with over 95% homology of major antigenic protein VP2, as compared to other AHSV-1 strains, including the prevalent strain in Thailand. In the mouse models, AHSV/C exhibited no clinical signs or histopathological lesions, suggesting low virulence and safety. This research for the first time characterized the in vitro growth characteristics and viral subtypes of the AHSV in China, determined its complete whole-genome sequence, and evaluated its safety using a mouse model. It provides crucial experimental materials and scientific foundations for the development of diagnostic methods and vaccines against AHSV-1.

## 1. Introduction

African horse sickness (AHS) is a non-contagious, arthropod-borne, infectious disease of equids caused by the African horse sickness virus (AHSV). This virus is a member of the genus Orbivirus of Sedoreovirinae subfamily within the family Reoviridae. The disease is endemic in sub-Saharan Africa, with regular spillovers into South Africa and occasional outbreaks in Northern Africa. Outbreaks of AHS have also occurred outside of Africa, including in the near and middle east regions, Spain, Portugal, the Cape Verde Islands, Thailand and Malaysia [1,2,3]. World Organization for Animal Health (WOAH) has listed this disease as a notifiable disease [4].

AHSV primarily infects equids, including horses, donkeys, mules and zebras via biting the midges of the Culicoides genus. Horse is the most susceptible, with a mortality rate of up to 90%. Mules and donkeys are less susceptible, with a mortality rate of 5% to 10%. Zebras are highly resistant to AHS and typically remain asymptomatic, thereby acting as natural reservoirs for AHSV. Some studies have shown the effects of AHSV on camels and other wildlife in nature [5,6,7,8]. In laboratory experiments, AHSV could infect mice and guinea pigs, which are frequently used for testing the safety of live-attenuated AHSV vaccines. Mice are also commonly used for isolation or assessment of the virulence of AHSV [9,10,11,12]. In vitro, a number of cell lines support AHSV propagation, such as African green monkey kidney (Vero), baby hamster kidney-21 (BHK-21), monkey stable (MS). All these cell lines are often used for isolation of AHSV and preparation of live-attenuated AHSV vaccines [9,13].

AHSV is a segmented, double-stranded, non-enveloped RNA virus. Its genome consists of ten RNA segments, encoding seven structural proteins (VP1-VP7) and six nonstructural proteins (NS1, NS2, NS3, NS3a, NS4I and NS4II). In this paper, each segment is named based on the protein it encodes. The NS3 and NS3a proteins are encoded by the same genome segment, named as ns3. VP6 is encoded by vp6 segment that also encodes NS4I and NS4II [14,15,16]. Currently, relatively little research has been conducted on the biological functions of viral proteins encoded by AHSV [16,17,18,19,20,21,22,23,24,25,26]. Among the limited studies available, the functions of key viral proteins have been identified: VP1 acts as an RNA-dependent RNA polymerase, while VP4 acts as a capping enzyme. VP6 acts as both a helicase and an ATPase, exhibiting activity in binding to both double-stranded and single-stranded RNA and DNA [27].These three viral enzyme proteins are located inside the virion and are encapsulated with viral genomic segments by core protein VP3, thus forming the sub-core particle. VP7, a major core protein, is deposited on the sub-core layer and forms the inner capsid to stabilize the viral sub-core. VP7 also plays a role in AHSV release and yield [17]. The VP7-encoding genomic RNA segment is highly conserved among all AHSV serotypes. Consequently, the VP7 gene and protein are commonly used as molecular and serological targets for diagnosis of AHSV [9,28]. VP2 and VP5 constitute the outer capsid of the virion and are both involved in cell attachment and entry. VP2 is the most variable antigen of AHSV, which contains most of the neutralizing epitopes of the virus [29,30,31]. VP5 is less exposed on the surface of the AHSV particles than VP2, and seems to play no role in AHSV neutralization [32]. The nonstructural proteins of virus are involved in regulating protein synthesis, as well as the assembly, transport, and release of AHSV from the infected cells [3].

Based on VP2 variability, AHSV can be classified into nine distinct serotypes (AHSV-1 to AHSV-9). These nine serotypes generally show minimal serological cross-reactivity. However, some cross-reaction has been observed between serotypes 1 and 2, 3 and 7, 5 and 8, and 6 and 9 [9]. AHSV-1 to AHSV-8 are found in limited areas and are considered highly pathogenic for horses, resulting in high mortality rates. On the contrary, AHSV-9 is widespread in endemic regions and appears to be less pathogenic, resulting in lower mortality [33,34,35,36]. In this study, an AHSV-1 strain imported to China from abroad in the 1960s was purified via plaque assays in Vero cells. In China, we characterized the in vitro growth properties and viral subtype of this strain for the first time, determined its complete genome sequence and evaluated its safety using a mouse model.

## 2. Materials and Methods

### 2.1. Virus Strain and Cell Culture

A strain of AHSV with little background information is stored at the National Center for Veterinary Culture Collection. This strain was imported into China in the 1960s and is a mouse brain tissue toxin that can be used as a vaccine candidate strain. In this study, this AHSV strain was passaged three times in a mouse brain and designated as AHSV/M. AHSV/M was passaged in African green monkey kidney cells (Vero cells) for plaque purification and designated as AHSV/C.

The Vero cells, provided by the National Center for Veterinary Culture Collection, were cultured in MEM medium at 37 °C with 5% CO_2_. The medium was supplemented with 8% heat-inactivated fetal bovine serum (FBS).

### 2.2. Virus Resuscitation

The freeze-dried AHSV strain was diluted using 10 mL of saline (0.9% sodium chloride injection) and then inoculated intracerebrally into specific pathogen-free (SPF) mice weighing 18–22 g, at a dose of 0.05 mL per mouse. The mice were housed in individually ventilated cages (IVC) with five mice in each cage. The clinical symptoms of the mice were observed daily, and the dead mice were frozen. The brain tissues of the sick and dead mice were taken after 14 days. These tissues were added into 10-fold volume of saline and then ground to prepare a virus solution. The virus was then passed through the mouse brains three times, using the same method, and was designated as AHSV/M.

To determine the median lethal dose (LD_50_) of AHSV/M, the viral solution was serially diluted 10-fold up to cell concentration of 10^−8^. Four dilutions (10^−5^ to 10^−8^) were selected and used for intracerebral inoculation of mice weighing 18–22 g, at a dose of 0.05 mL per mouse. A normal saline control group was set up simultaneously. The clinical symptoms of mice were observed continuously for 14 days, and the LD_50_ was calculated using the Reed-Muench method.

### 2.3. AHSV/M Adaptation to Cell Culture

In order to obtain cell-adapted strains, AHSV/M was diluted tenfold in cell culture medium, inoculated into a monolayer of Vero cells and continuously passaged. The presence of cytopathic effect (CPE) in the cells was assessed daily. If no CPE was observed within 120 h, a blind passage was performed. Once 80% CPE was achieved, the virus was harvested and freeze-thawed repeatedly three times. The virus was then inoculated onto fresh Vero cells for passage again. The harvested virus solution was stored in a −70 °C refrigerator for later use.

### 2.4. Virus Titration

The harvested virus solution was serially diluted tenfold. The appropriate dilutions were inoculated into 96-well cell culture plates with confluent monolayers, using one dilution for 5 wells. The non-inoculated cells were set as the negative controls. Each well was inoculated with 0.1 mL of virus solution, and 0.1 mL of cell maintenance solution was added. The culture plates were incubated at 37 °C with 5% CO_2_ for 120 h, and cell CPEs were observed. TCID_50_ was calculated by the Reed-Muench method.

### 2.5. Plaque Purification

In this study, plaque purification of AHSV was performed as described previously [37]. After serially diluting the 12th passage cell-adapted AHSV/M, two dilutions (10^5^ and 10^6^) were used for infecting the Vero cells, using 0.5 mL dilution per dish. After adsorption at 37 °C for 1 h, the liquid was discarded and the dishes were filled with 1% agar medium. The dishes were incubated at 37 °C with 5% CO_2_. Once CPEs were visible under the microscope, a layer of neutral red agar was added, followed by overnight incubation. The next day, single clones with larger plaques that were far from the other plaques were picked and transferred into 0.5 mL of cell maintenance solution. The solution was freeze-thawed three times, centrifuged and then the supernatant was inoculated into cell culture dishes containing confluent monolayers. Cloning and purification were performed for six passages by following the above steps.

### 2.6. Determination of Virus Growth Curve

The AHSV/C virus solution was diluted to cell concentrations of 10^−3^ and 10^−4^. Then, 0.5 mL of each diluted solution was transferred into a culture flask (75 cm^2^) containing a continuous cell monolayer. To observe the CPEs, 0.5 mL of culture sample was collected every 24 h for five days and stored at −70 °C. Finally, the viral titer in each sample was determined.

### 2.7. RT-PCR (Reverse Transcription Polymerase Chain Reaction)

Referring to the RT-PCR method recommended by WOAH in the Manual of Diagnostic Tests and Vaccines for Terrestrial Animals, the conserved VP7 gene fragment of AHSV was amplified, and mRNAs extracted from uninfected Vero cells served as the negative control. The primer sequences used for amplification were 5′-GTT-AAA-ATT-CGG-TTA-GGA-TG-3′ and 5′-GTA-AGT-GTA-TTC-GGT-ATT-G-3′. The reaction cycle for one-step RT-PCR was as follows: 50 °C for 50 min; 95 °C for 10 min; then 40 cycles of 95 °C (1 min), 55 °C (1 min), and 72 °C (2 min); and a final extension at 72 °C for 8 min [9]. PCR products were analyzed by 1% agarose gel electrophoresis and then sequenced.

### 2.8. Virus Neutralization Test

AHSV/C virus was diluted in cell culture medium to obtain a concentration of 100 TCID_50_/0.1 mL. 1 mL of this diluted virus solution was mixed with an equal volume of AHSV-1-positive horse serum (provided by the Pribright Institute, an AHS reference laboratory of WOAH) and incubated at 37 °C for 1 h. The solution was then inoculated into 5 wells of a 96-well plate containing Vero cells, with 0.1 mL solution per well. Meanwhile, 5 wells of positive control and 5 wells of negative control were set up. In the positive control wells, diluted virus solution was mixed with an equal volume of cell culture medium for inoculation. In the negative wells, Vero cells were inoculated with only the cell culture medium. The plates were incubated at 37 °C and 5% CO_2_ level for 120 h, and the number of wells showing CPE in each group was observed.

### 2.9. IFA (Immunofluorescence Assay)

In this study, immunofluorescence assay was performed as described previously [38]. Briefly, AHSV/C was serially diluted 10-fold with serum-free cell culture medium to a concentration of 10^−4^. The diluted virus solution was inoculated into a 96-well plate with confluent Vero cells, at a dose of 0.1 mL per well. The virus-free wells were set as the controls. After 48 h, the cell culture medium was discarded, and the cell surface was washed once with phosphate-buffered saline (PBS). After removing the PBS, cold methanol was added to fix the cells. The methanol was discarded after 15 min, and the plates were air-dried. Subsequently, 10-fold diluted AHSV-1-positive horse serum was added to the plates and the plates were incubated at 37 °C for 1 h (AHSV-1-negative horse serum were set as negative controls). After 1 h, the serum was discarded, and the cells were washed 3 times with PBS containing 0.05% Tween-20, followed by 2 washes with PBS. After removing all the liquids, 500-fold diluted rabbit anti-horse IgG H&L (FITC conjugated) was added and the plates were incubated at 37 °C for 1 h. After incubation, the cells were washed again as mentioned above. The presence of specific green fluorescence was observed under an inverted fluorescence microscope (OLYMPUS, IX71).

### 2.10. Whole-Genome Sequencing and Analysis

RNA of AHSV/C was extracted using a virus DNA/RNA extraction kit (Tiangen Biotech Co., Ltd., Beijing, China. Cat.#DP315). Total RNA from the sample was sequenced using the paired-end (PE) strategy on the Illumina platform. The generated next-generation sequencing data were assembled with SPAdes (v3.14.1) and SOAPdenovo (Version 2.04) software. Depth statistics were performed on the assembled contigs file (i.e., the coverage of reads on contigs) to verify the accuracy and coverage of the reads relative to the assembly results, with only contigs ≥1500 bp in length selected for the statistics. Finally, the Prokka software (V 1.14.5) was used to conduct gene functional annotation of the assembly results. The homology of the obtained sequences with reference sequences published in GenBank was analyzed. A phylogenetic tree, based on VP2 gene, was constructed using the maximum likelihood method and Tamura-Nei model of MEGA 11. The tree with the highest log likelihood (−38,658.34) is shown. A discrete Gamma distribution was used to model evolutionary rate differences among sites (5 categories (+G, parameter = 2.0832)). The percentage of replicate trees in which the associated taxa clustered together in the bootstrap test (1000 replicates) is shown. Evolutionary distances were computed via the Kimura 2-parameter method and are expressed as the number of base substitutions per site. Only bootstrap values > 70% are displayed. A reassortment analysis was performed using the Recombination Detection Program (RDP) to clarify the relationship between AHSV/C and AHSV-1 isolate 1180 (GenBank accession numbers KP009711-KP009720), the AHSV-2 strain (AHSV-2_82_61_LP) (GenBank accession numbers KY471471, KY471479, KY471487, KY471495, KY471503, KY471511, KY471519, KY471527, KY471535 and KY471543), and the AHSV-8 strain (8/Labstr/ZAF/1998/OBP-252.1) (GenBank accession numbers KT715631-KT715640), based on the corresponding concatenated whole virus genomes [39,40].

### 2.11. Safety Evaluation of AHSV/C in a Mouse Model

To determine the safety of AHSV/C in a mouse model, 40 female SPF Balb/C mice weighing 18–22 g were selected and divided into four groups of 10 mice. The mice of three groups were injected with AHSV/C at a titer of 10^7.5^ TCID_50_/mL, 10^6.5^ TCID_50_/mL, and 10^5.5^ TCID_50_/mL (TCID_50_ represents 50% tissue culture infectious dose), while the fourth group (control group) was injected with saline. According to the inoculation method recommended by WOAH for AHS safety evaluation [9], each mouse was intraperitoneally injected with 0.25 mL. The following indicators were evaluated for the mice:

Clinical symptoms: the clinical symptoms of the mice were observed daily for 14 consecutive days.

Weight gain: We defined the day before virus infection as Day 0 (0 dpi, where dpi refers to days post-infection), and the body weight of each mouse measured on this day was set as the “baseline body weight”. For each subsequent time point (1 dpi, 4 dpi, 7 dpi, 10 dpi, 14 dpi), the changes in body weight of each mouse was calculated using the formula: Changes in body weight of each mouse (g) = Body weight at the target dpi (g)—Baseline body weight (0 dpi, g). Independent samples t-test was applied to compare the body weight changes between the inoculated group and the control group (statistical significance was calculated using SPSS software 26.0, n = 10).

Histopathological examination: On 14 dpi, 2 mice from each group and 1 mouse from the control group were necropsied. Gross pathological changes in various tissues and organs were evaluated. The pathological sections of heart, liver, spleen, lung, kidney, and brain of mice were prepared at Wuhan Sevier Biotechnology Co., Ltd. (Wuhan, China) for histopathological examination.

## 3. Results and Discussion

### 3.1. Characterization of AHSV/C Strain

**Figure 1:**
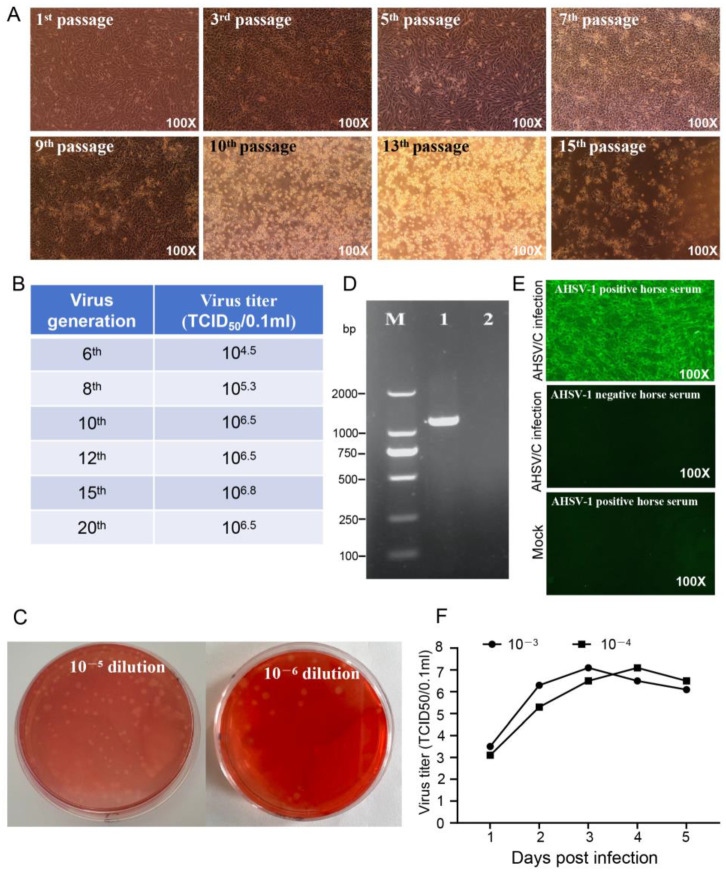
Adaptability and identification of AHSV/C in Vero cells: (**A**). CPE induced by indicated passages of AHSV/M during serial propagation in Vero cells, with 1st passage to 15th passage representing the number of passages of AHSV/M in Vero cells; (**B**). Virus titer of different passages of AHSV/M during serial propagation in Vero cells (The experiment was repeated two times, and the results of the two trials were similar); (**C**). After 12 generations of passage, AHSV/M formed primary plaques in Vero cells (The left panel shows a 10^−5^ dilution, and the right panel shows a 10^−6^ dilution); (**D**). RT-PCR identification of AHSV/C based on VP7 genomic fragment (M: DL2000 marker; 1: mRNA extracted from Vero cells infected with AHSV/C; and 2: mRNA extracted from the uninfected Vero cells as a negative control); (**E**). Identification of AHSV/C using IFA with an AHSV-1-positive/negative horse serum (The experiment was repeated two times, and the results of the two trials were similar); Mock: uninfected Vero cells; (**F**). The growth curve of AHSV/C, with 10^−3^ and 10^−4^ indicating the dilutions of seeded AHSV/C, respectively (The experiment was repeated two times, and the results of the two trials were similar).

To determine the biological characteristics of the AHSV used in this study, the virus was inoculated intracerebrally into mice and was continuously propagated in the mice for three generations. At 72 h post inoculation, the mice gradually began to exhibit clinical symptoms, such as depression, arched backs and limb convulsions, eventually dying. Autopsy revealed severe bleeding in the brain tissue, the strain was designated AHSV/M. The titer of AHSV/M for mice was determined to be 10^7.8^ LD_50_/0.1 mL. Subsequently, the AHSV/M was subjected to blind passages in Vero cells. The CPEs became observable starting from the 5th passage and stabilized by the 10th passage (Figure 1A). The viral titers from the 10th to 20th passages were approximately 10^6.5^ TCID_50_/0.1 mL (Figure 1B). A total of six rounds of plaque purification (Figure 1C) were performed for the twelfth-generation virus. The VP7 gene-specific RT-PCR recommended by the WOAH [8] (Figure 1D) and indirect immunofluorescence (IFA) (Figure 1E) were performed to confirm that the purified virus was AHSV [8]. This version of the strain was designated AHSV/C. Furthermore, a virus neutralization (VN) test, which is the gold standard for serotyping AHSV isolates [8], was performed to confirm that the AHSV/C belongs to AHSV-1. The multistep growth curves of AHSV/C in Vero cells were measured, and the results showed that the viral titer reached a maximum value of 10^7.1^ TCID_50_/0.1 mL (Figure 1F).

### 3.2. Genomic Characterization and Phylogenetic Analysis of AHSV/C

To further analyze the genomic characteristics of AHSV/C, Illumina Novaseq6000 PE150 sequencing technology was used. The complete genome sequence of AHSV/C has been uploaded to GenBank. Table 1 shows the accession number, sequence size, coding sequence size of each AHSV/C genomic segment, the percent identity with AHSV-1 isolate 1180 and Thailand 2020 strain (TAI2020/01).

**Figure 2:**
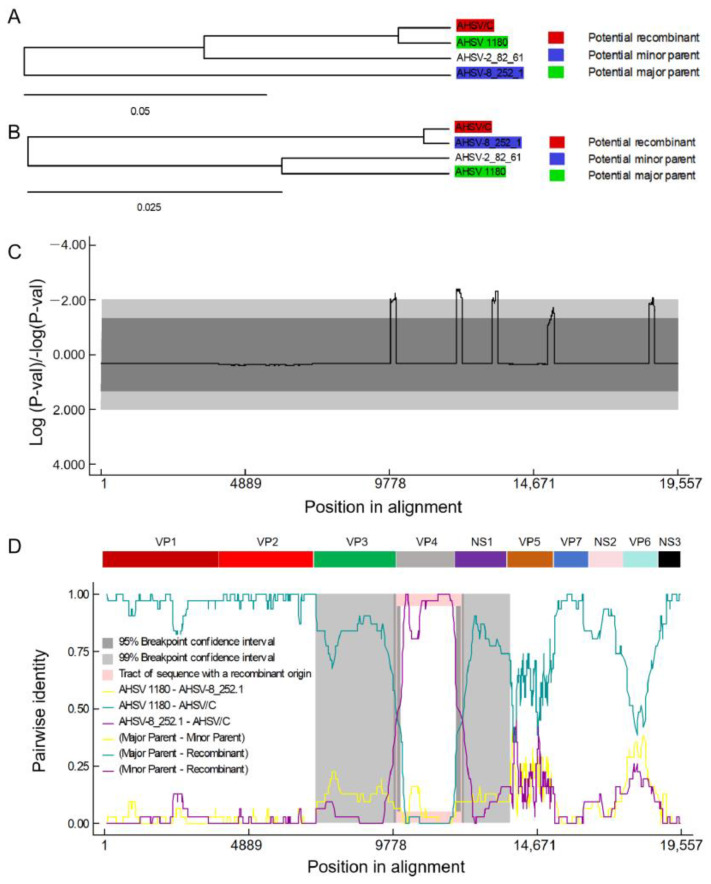
Identification of AHSV/C genome reassortment events. (**A**) Unweighted Pair-Group Method with Arithmetic mean (UPGMA) phylogenetic tree of nucleotides 1–9909 and 12,137–19,557 of concatenated whole-virus genome, (**B**) UPGMA phylogenetic tree of nucleotides 9910–12,136 of concatenated whole-virus genome, (**C**) Breakpoint distribution and (**D**) Reassortment detection on the basis of pairwise distance identity.

BLAST (2.17.0) analysis of all ten AHSV/C genomic fragments revealed that eight of these segments (encoding VP1, VP2, VP3, VP6, VP7, NS1, NS2 and NS3) exhibited the highest homology with the corresponding genomic fragments of a neurotropic AHSV-1 strain (isolate 1180). This isolate was discovered in South Africa in 1933 and has been passaged over 100 generations in adult mice [41]. The respective similarity levels of these nucleotide segments were 99.74%, 98.97%, 99.32%, 97.95%, 99.57%, 99.37%, 99.31% and 97.27% (Tables S1–S8). Furthermore, the genomic fragment encoding VP4 of AHSV-C exhibited the highest homology with an AHSV-8 (8/Labstr/ZAF/1998/OBP-252.1) strain isolated in South Africa in 1998, with an identity level of 99.49%. Whereas the percent identity of VP4 segment with the corresponding fragment of isolate 1180 was only 91% (Table S9). The genomic fragment encoding VP5 of AHSV/C exhibited the highest homology with the corresponding genomic fragments of an AHSV-2 strain isolated in South Africa in 1961 (AHSV-2_82_61_LP), with percent identity of 96.74%, whereas the percent identity with isolate 1180 was only 84.65% (Table S10). To clarify the relationship between AHSV/C and AHSV-1 isolate 1180, AHSV-2 (AHSV-2_82_61_LP), AHSV-8 (8/Labstr/ZAF/1998/OBP-252.1), the study conducted a reassortment analysis of the concatenated full viral genomes of these four strains using RDP software. As shown in Figure 2, AHSV/C showed the strongest association with AHSV-1 (strain 1180), with four of its genomic segments (encoding VP1, VP2, VP7, and NS3) sharing similarity with those of AHSV-1 (strain 1180). Meanwhile, one genomic segment of AHSV/C (encoding VP4) exhibited similarity to that of AHSV-8 (8/Labstr/ZAF/1998/OBP-252.1). Additionally, no reassortment association or significant sequence inheritance relationship was observed between AHSV/C and AHSV-2_82_61_LP.

In 2020, an outbreak of AHS caused by AHSV-1 occurred in Thailand [2,42]. Therefore, the percent identities of the genome of AHSV/C used in this study was compared to the AHSV-1 strain isolated in Thailand in 2020 (TAI2020/01). The identity levels of AHSV/C segments were as follows: VP1-encoding fragment (88.9%), VP2-encoding fragment (95.28%), VP3-encoding fragment (95.27%), VP4-encoding fragment (89.07%), VP5-encoding fragment (95.46%), VP6-encoding fragment (95.38%), VP7-encoding fragment (96.39%), NS1-encoding fragment (96.85%), NS2-encoding fragment (95.18%) and NS3-encoding fragment (95.54%) (Tables S1–S10). The above information indicates that the two virus strains are closely related, with their core antigenic genes and functional genes being highly conserved.

**Figure 3:**
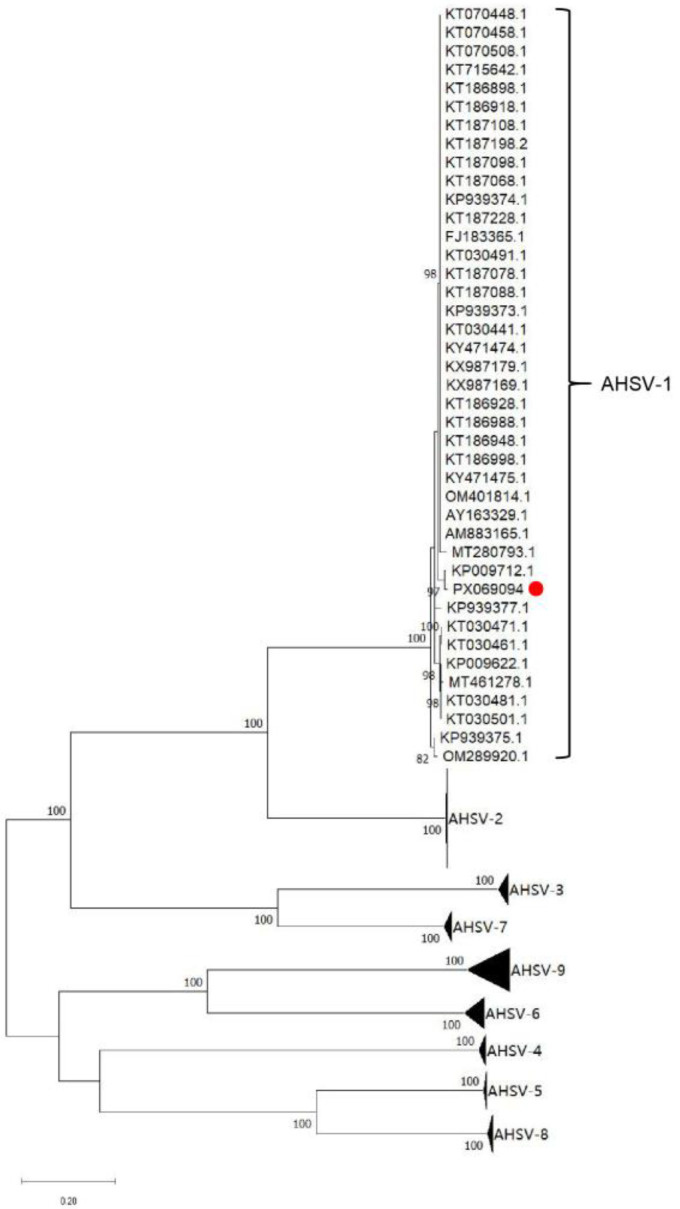
A phylogenetic tree constructed using MEGA 11, based on the complete *vp2* gene sequence from AHSV-1 to AHSV-9 (The red dot represent the AHSV/C *vp2* sequence, The accession number of the Thailand 2020 strain (TAI2020/01) is MT461278.1).

VP2 of AHSV is known as a major protective antigen recognized by serotype-discriminatory neutralizing antibodies [3,29,43,44]. A phylogenetic analysis, based on the complete open reading frame (ORF) sequences of VP2 gene from AHSV serotypes 1–9, was conducted. As shown in Figure 3, the VP2 sequence of AHSV/C clusters and the VP2 sequences of AHSV-1 were observed on the same branch, which confirmed that AHSV/C belonged to AHSV-1. Furthermore, a multiple sequence alignment was performed, and the results showed that VP2 amino acid sequences of 40 AHSV serotype 1 isolates were highly conserved (Figure S1). The percentage identity of amino acid sequences between AHSV/C VP2 and other AHSV-1 VP2 ranged from 95.9% to 98.1%. In previous studies, neutralizing epitopes were identified in AHSV-4 VP2 [30,34]. However, due to significant divergence among the VP2 proteins of different AHSV serotypes, the neutralizing epitopes present in AHSV-4 VP2 was absent in AHSV-1 VP2. It is worth noting that the two conserved linear epitopes in the AHSV-1 VP4, recently identified by mouse antibodies [38], were also present in AHSV/C VP4 (Figure S1).

### 3.3. Safety Evaluation of AHSV/C in a Mouse Model

**Figure 4:**
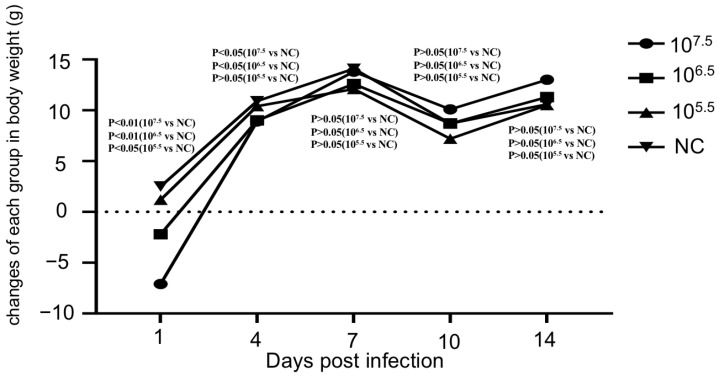
The curves graph showing the changes in the body weight of mice infected with different doses of AHSV/C (10^7.5^, 10^6.5,^ and 10^5.5^ indicate the viral doses administered to AHSV/C-infected mouse groups; NC denotes the control group of mice injected with saline solution; The *p*-value was calculated via the independent samples t-test using SPSS software. *p*-value < 0.01 indicates an extremely significant statistical difference in weight gain between mice in the immune group and those in the control group, *p*-value < 0.05 indicates a significant statistical difference in weight gain between mice in the immune group and those in the control group, *p*-value > 0.05 indicates no significant statistical difference in weight gain between mice in the immune group and those in the control group. n = 10); B. Microscopic features of the lung, liver, brain, spleen, kidney and heart of mice infected with different doses of AHSV/C (Representative images are shown for each experimental group).

Currently, there is no ideal animal model for AHSV. However, some studies have suggested that mouse model may be effective in evaluating the virulence of AHSV [12,36]. To evaluate the safety of AHSV/C, three groups of mice were inoculated intraperitoneally with 0.25 mL of AHSV/C at three different titers (10^7.5^, 10^6.5^ and 10^5.5^ TCID_50_/_mL_) and observed daily for 14 days, according to the method recommended by WOAH for assessing the safety of AHSV vaccines [9]. Throughout the entire observation period, no obvious clinical symptoms were observed in mice in either the infected groups or the mock-infected (control) group. An independent samples t-test was used to analyse body weight changes (Figure 4) (with significance calculated using SPSS software). The results revealed a positive correlation between the inoculation dose and the intensity of early body weight changes in mice. On 1 dpi, the body weight of mice in all infected groups decreased significantly compared to the control group (*p* < 0.01 for the high-dose group, 10^7.5^ TCID_50_/mL; *p* < 0.01 for the medium-dose group, 10^6.5^ TCID_50_/mL; and *p* < 0.05 for the low-dose group, 10^5.5^ TCID_50_/mL). Subsequently, the body weight of mice in all groups gradually resumed an increasing trend, on 4 dpi, there was no significant difference in body weight gain between the low-dose group and the control group (*p* > 0.05); On 7 dpi and until the end of the observation period, no statistically significant differences in body weight gain were observed between any of the infected groups and the control group (*p* > 0.05). It is noteworthy that all mice (including those in the control group) experienced body weight loss on 10 dpi. This phenomenon is not directly related to viral infection (as the control group had no exposure to the virus) and is presumably caused by non-infectious interfering factors (such as environmental stress, fluctuations in feeding conditions, or operation-related stress).

**Figure 5:**
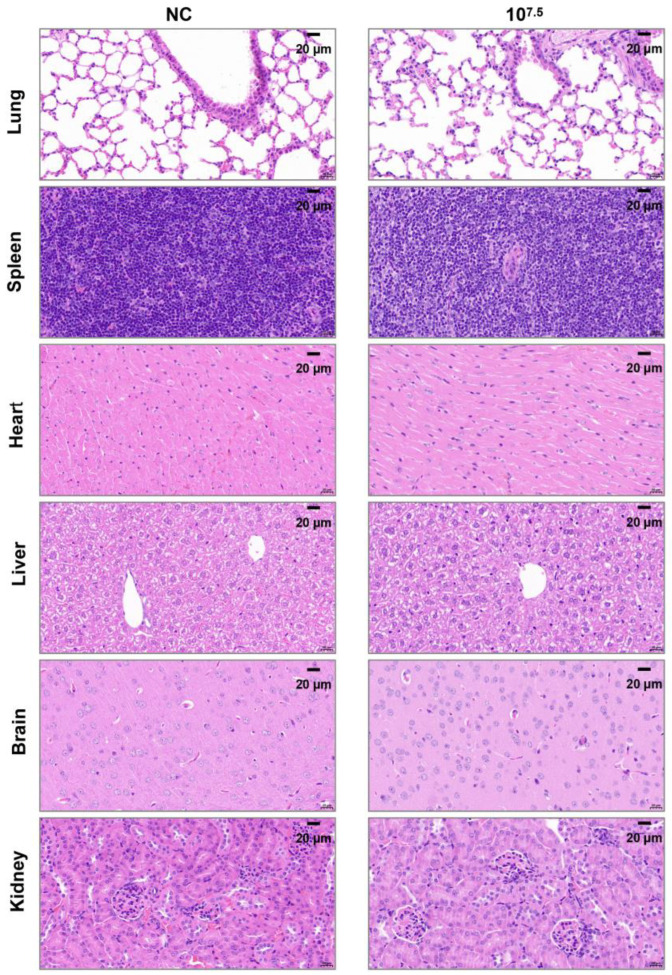
Microscopic features of the lung, liver, brain, spleen, kidney and heart of mice infected with AHSV/C at a dose of 10^7.5^ TCID_50_/mL, as shown by HE staining.

Autopsies were conducted on mice from both the infected and control groups, and no obvious gross pathological lesions were observed. Furthermore, no pathological lesions were observed in any of the infected mice during histopathological examinations (Representative images are shown in Figure 5 for the high-dose group, 10^7.5^ TCID_50_/mL. The images of the medium-dose group and the low-dose group are shown in the supplementary original images file).

Collectively, these results indicate that AHSV/C exhibits low pathogenicity in mice. However, to fully assess the virulence of AHSV/C, further evaluations in horses, the natural hosts of AHSV, are required.

In 2020, an AHS outbreak caused by AHSV-1 in Thailand posed a substantial threat to the neighboring nations [2,42,45]. To effectively address the potential risks posed by such outbreaks and prevent their occurrence, it is crucial to intensify the research on AHSV-1, including the development of detection methods and vaccines and investigations into the underlying mechanisms. To ensure the emergency reserve for AHS prevention and control, AHSV strain imported to China from abroad over 60 years ago was characterized in this study. The original virus was a mouse brain-derived strain, which required cultivation in live mice. This culture method is not only complex in operation but also limited by the use of live animals, making it unfavorable for large-scale propagation and systematic identification of the virus. To address this, we gradually adapted the virus to an in vitro cell culture system, and after cloning and purification, successfully obtained a stable cell-adapted virus strain, providing high-quality experimental materials for subsequent research. On this basis, we further conducted biological characterization and whole-genome sequencing analysis of this cell-adapted virus, and the results confirmed that it is an AHSV-1 strain.

Whole-genome sequencing revealed that AHSV/C was highly homologous to AHSV-1 isolate 1180, with over 95% homology of major antigenic protein VP2, as compared to other AHSV-1 strains, including the prevalent strain in Thailand. Consequently, there is an opportunity to use AHSV/C and its VP2 to develop AHSV-1 candidate vaccines that could elicit cross-protective immunity against the circulating strains of AHSV-1, including those that have caused recent outbreaks. Meanwhile, we also conducted a recombination analysis to determine the relationship between AHSV/C and AHSV-1 isolate 1180, AHSV-2 (AHSV-2_82_61_LP), AHSV-8 (8/Labstr/ZAF/1998/OBP-252.1). The results confirmed that AHSV/C has the strongest association with AHSV-1 (strain 1180), with four of its genomic segments (encoding VP1, VP2, VP7, and NS3) sharing similarity with those of AHSV-1 (strain 1180). Meanwhile, one genomic segment of AHSV/C (encoding VP4) exhibited similarity to that of AHSV-8 (8/Labstr/ZAF/1998/OBP-252.1). Additionally, no reassortment association or significant sequence inheritance relationship was observed between AHSV/C and AHSV-2_82_61_LP. By integrating the key information of “virus isolation date” (8/Labstr/ZAF/1998/OBP-252.1 was isolated later than AHSV/C), the possibility that “8/Labstr/ZAF/1998/OBP-252.1 serves as the parental strain of AHSV/C” can be ruled out. It is hypothesized that the similarity of their VP4 segments may originate from other common ancestral viruses, or that this segment of AHSV-8 was inherited from an earlier virus related to AHSV/C. A study by Guthrie et al. showed that 8/Labstr/ZAF/1998/OBP-252.1 is a reassortant with the segment encoding VP4 likely derived from an AHSV for which whole-genome sequence data are not available [40]. Reassortment is a common feature of AHSV, representing a major contributing factor in the genetic diversity of field strains, and often causes outbreaks of novel circulating strains.

Safety evaluation in mice showed that AHSV/C has low pathogenicity to mice and exhibits good safety in this mouse model. This finding provides an experimental basis for the preliminary safety assessment of AHSV/C—mice are often used as an experimental surrogate model in early viral pathogenicity studies due to their low cost and easy operation. However, the equids are the natural hosts of AHSV and possess distinct susceptibility and pathological responses from those of mice. Conclusions drawn solely based on the mouse model may not fully reflect the actual epidemiological risk and pathogenic potential of AHSV/C in horse populations. Therefore, further studies are still needed to evaluate the pathogenicity of AHSV/C to horses (the main target host for AHSV prevention and control). Similar insights have also been emphasized in recent studies on the pathogenesis of lumpy skin disease virus (LSDV) in indigenous cattle, where Kutumbetov et al. (2025) confirmed the importance of evaluating strain-specific virulence and host responses in regional contexts [46]. Their study further pointed out that regional differences in the host genetic background may alter the expression of viral virulence—a finding that also holds implications for AHSV, underscoring the necessity of conducting pathogenicity studies on AHSV in its natural hosts and regionally relevant populations. This commonality not only highlights the limitations of current research findings based on mouse models but also provides guidance for designing subsequent studies.

As an AHS-free country, China has taken proactive measures to address the potential threats of disease occurrence. But historical data on AHSV has long been limited—most studies have focused on theoretical risk assessments rather than empirical analysis of actual strains. These research findings enrich the currently limited number of full-length AHSV-1 genomes publicly available, and the cell-adapted strain (AHSV/C) obtained in this study also provided crucial experimental materials and scientific foundations for developing diagnostic methods and vaccines for AHSV-1.

## Figures and Tables

**Table 1:** Genomic information of AHSV/C.

Genome Segment	mRNA Length	CDS Length	GenBank Accession No.	Percent Identity with AHSV-1 Isolate 1180 ^f^ (%)	Percent Identity with Thailand 2020 Strain (TAI2020/01) ^g^ (%)
Segments encoding VP1	3965	3918 (14–3931)	PX069092	99.47	88.9
Segments encoding VP2	3218	3171 (13–3183)	PX069094	98.97	95.28
Segments encoding VP3	2792	2718 (27–2744)	PX069096	99.32	95.27
Segments encoding VP4	1978	1929 (12–1940)	PX069098	91.00	89.07
Segments encoding VP5	1564	1518 (20–1537)	PX0690100	84.65	95.46
Segments encoding VP6	1169	1110 (18–1127) ^a^ 240 (196–435) ^b^ 288 (148–435) ^c^	PX0690102	97.95	95.38
Segments encoding VP7	1167	1050 (18–1067)	PX0690104	99.57	96.39
Segments encoding NS1	1748	1647 (36–1682)	PX0690106	99.37	96.85
Segments encoding NS2	1166	1098 (23–1120)	PX0690108	99.31	95.18
Segments encoding NS3	763	657 (19–675) ^d^ 627 (49–675) ^e^	PX0690110	97.27	95.54

Note: a, indicates the CDS encoding VP6; b, indicates the CDS encoding NS4I; c, indicates the CDS encoding NS4II; d, indicates the CDS encoding NS3; e, indicates the CDS encoding NS3a. f, The GenBank accession numbers for the ten genomic segments of AHSV-1 isolate 1180 are KP009711-KP009720; g, The GenBank accession numbers for the ten genomic segments of the Thailand 2020 strain are MT586213-MT586221 and MT461278.

## Data Availability

All required data are available in the manuscript. Any additional data can be provided upon request.

## Supplementary Materials

The following supporting information can be downloaded at: https://www.mdpi.com/article/10.3390/microorganisms13102314/s1, Figure S1: Comparison of the deduced amino acid sequences of AHSV-1 VP2; Table S1: Blast N search results for vp1 segment of the AHSV/C: Table S2: Blast N search results for vp2 segment of the AHSV/C; Table S3: Blast N search results for vp3 segment of the AHSV/C; Table S4: Blast N search results for vp6 segment of the AHSV/C; Table S5: Blast N search results for vp7 segment of the AHSV/C; Table S6: Blast N search results for ns1 segment of the AHSV/C; Table S7: Blast N search results for ns2 segment of the AHSV/C; Table S8: Blast N search results for ns3 segment of the AHSV/C; Table S9: Blast N search results for vp4 segment of the AHSV/C; Table S10: Blast N search results for vp5 segment of the AHSV/C.

## Abbreviations

The following abbreviations are used in this manuscript:

AHSV	African horse sickness virus
AHS	African horse sickness
RT-PCR	Reverse transcription polymerase chain reaction
LD_50_	Lethal dose, 50%
TCID_50_	50% tissue culture infectious dose
PBS	Phosphate-buffered saline
