# Supplementary material for: Identification and Genetic Characterization of a Strain of African Horse Sickness Virus Serotype 1 and Its Safety Evaluation in a Mouse Model
Source: Microorganisms. 2025 Oct 6;13(10):2314. doi: 10.3390/microorganisms13102314 (PMC12566010; doi:10.3390/microorganisms13102314)
Supplement: Supplementary file 1 [file microorganisms-13-02314-s001.zip › Figure S1.pdf]

Figure S1, to be continued-1

```
                      *        20         *        40         *        60         *        80         *       100
PX069094   : MASEFGILLTERIFDETLEKTNCDVIITEEKKVKRKEVEGVLGYVWEETNHRFGLCEGNYDLAFSDTMYRQTRYDGAYPVFPHYIIDALRYGVMIDRNDN : 100
KP939375.1 : .....................................................................C...C.......................... : 100
OM289920.1 : .....................................................................C...C.......................... : 100
KT030471.1 : ..................................................................A..C...C.......................... : 100
KT030461.1 : ..................................................................A..C...C.......................... : 100
KT030501.1 : ..................................................................A..C...C.......................... : 100
KT030481.1 : ..................................................................A..C...C.......................... : 100
KP009622.1 : ..................................................................A..C...C.......................... : 100
MT461278.1 : ..................................................................A..C...C.......................... : 100
KP939377.1 : .....................................................................C.I.C.......................... : 100
KP009712.1 : .....................................................................Y...C.......................... : 100
KT030441.1 : .....................................................................C...C..S....................... : 100
KP939373.1 : .....................................................................C...C..S....................... : 100
KT187088.1 : .....................................................................C...C..S....................... : 100
FJ183365.1 : .....................................................................C...C..S....................... : 100
KY471474.1 : .....................................................................C...C..S....................... : 100
KT187068.1 : .....................................................................C...C..S....................... : 100
KT030491.1 : .....................................................................C...C..S....................... : 100
KT187228.1 : .....................................................................C...C..S....................... : 100
KP939374.1 : .....................................................................C...C..S....................... : 100
KT070448.1 : ..................................................................A..C...C..S....................... : 100
KT070458.1 : ..................................................................A..C...C..S....................... : 100
KT070508.1 : ..................................................................A..C...C..S....................... : 100
KT187108.1 : .....................................................................C...C..S....................... : 100
KT187098.1 : .....................................................................C...C..S....................... : 100
KT187198.2 : .....................................................................C...C..S....................... : 100
KT186898.1 : .....................................................................C...C..S....................... : 100
KT715642.1 : .....................................................................C...C..S....................... : 100
KT186918.1 : .....................................................................C...C..S....................... : 100
KT187078.1 : .....................................................................C...C..S....................... : 100
KT186988.1 : .....................................................................C...C..S....................... : 100
KX987169.1 : .....................................................................C...C..S....................... : 100
KT186998.1 : .....................................................................C...C..S....................... : 100
KT186948.1 : ......................................K..............................C...C..S....................... : 100
KT186928.1 : .....................................................................C...C..S....................... : 100
AY163329.1 : ..................G..................................................C...C..S....................... : 100
KX987179.1 : .....................................................................C...C..S....................... : 100
AM883165.1 : .....................................................................C...C..S....................... : 100
KY471475.1 : .....................................................................C...C..S....................... : 100
OM401814.1 : .....................................................................C...C..S....................... : 100
```

Figure S1, to be continued-2

```
                          *       120         *       140         *       160         *       180         *       200
PX069094   : QVRVDLDDKRLMKIKIQPYMGEMYFSPENYSTVFCKRQALALGVDDLRHSVDVRNEFEETTHQTRGVLNGNKLRALEVWKEMAYQRMRKEGSRGRCIGHD : 200
KP939375.1 : ............................................................N...........Q........................... : 200
OM289920.1 : ............................................................N...........Q........................... : 200
KT030471.1 : ............................................................N........A..Q........................... : 200
KT030461.1 : ............................................................N........A..Q........................... : 200
KT030501.1 : ............................................................N...........Q........................... : 200
KT030481.1 : ............................................................N...........Q........................... : 200
KP009622.1 : ............................................................N...........Q........................... : 200
MT461278.1 : ............................................................N....S......Q........................... : 200
KP939377.1 : ................................................R...........N...........Q........................... : 200
KP009712.1 : ............................................................I....................................... : 200
KT030441.1 : ............................................................N...........Q........................... : 200
KP939373.1 : ............................................................N...........Q........................... : 200
KT187088.1 : ............................................................N...........Q........................... : 200
FJ183365.1 : ............................................................N...........Q........................... : 200
KY471474.1 : ............................................................N...........Q........................... : 200
KT187068.1 : ............................................................N...........Q........................... : 200
KT030491.1 : ............................................................N...........Q........................... : 200
KT187228.1 : ............................................................N...........Q........................... : 200
KP939374.1 : ............................................................N...........Q........................... : 200
KT070448.1 : ............................................................N...........Q........................... : 200
KT070458.1 : ............................................................N...........Q........................... : 200
KT070508.1 : ............................................................N...........Q........................... : 200
KT187108.1 : ............................................................N...........Q........................... : 200
KT187098.1 : ............................................................N...........Q........................... : 200
KT187198.2 : ............................................................N...........Q........................... : 200
KT186898.1 : ............................................................N...........Q........................... : 200
KT715642.1 : ............................................................N...........Q........................... : 200
KT186918.1 : ............................................................N...........Q........................... : 200
KT187078.1 : ............................................................N...........Q........................... : 200
KT186988.1 : ............................................................N...........Q........................... : 200
KX987169.1 : ............................................................N...........Q........................... : 200
KT186998.1 : ............................................................N...........Q........................... : 200
KT186948.1 : ............................................................N...........Q........................... : 200
KT186928.1 : ............................................................N...........Q........................... : 200
AY163329.1 : ............................................................N...........Q..........H................ : 200
KX987179.1 : ............................................................N...........Q........................... : 200
AM883165.1 : ............................................................N...........Q........................... : 200
KY471475.1 : ............................................................N...........Q........................... : 200
OM401814.1 : ............................................................N...........Q........................... : 200
```

```
                   *        220         *        240         *        260         *        280         *        300
PX069094    : DDVMYQLIKKLRFGMMYPHHYALNTRYEVSNPSAARIKDWLLKVRVNVGRAQEKADQTGPLAEMARSIENDELSRQVVDQIIQYGGQFSSCSGTREDDIP : 300
KP939375.1  : ........................A....................................................................A...... : 300
OM289920.1  : ........................A................................................N...................A...... : 300
KT030471.1  : ........................A........T...........................................................A...... : 300
KT030461.1  : ........................A........T...........................................................A...... : 300
KT030501.1  : ........................A....................................................................A...... : 300
KT030481.1  : ........................A....................................................................A...... : 300
KP009622.1  : ........................A....................................................................A...... : 300
MT461278.1  : ........................A....................................................................A...... : 300
KP939377.1  : ........................A....................................................................A...... : 300
KP009712.1  : ................................G................................................................... : 300
KT030441.1  : ........................A....................................................................A...... : 300
KP939373.1  : ........................A....................................................................A...... : 300
KT187088.1  : ........................A....................................................................A...... : 300
FJ183365.1  : ........................A....................................................................A...... : 300
KY471474.1  : ........................A....................................................................A...... : 300
KT187068.1  : ........................A....................................................................A...... : 300
KT030491.1  : ........................A....................................................................A...... : 300
KT187228.1  : ........................A....................................................................A...... : 300
KP939374.1  : ........................A....................................................................A...... : 300
KT070448.1  : ........................A....................................................................A...... : 300
KT070458.1  : ........................A....................................................................A...... : 300
KT070508.1  : ........................A....................................................................A...... : 300
KT187108.1  : ........................A....................................................................A...... : 300
KT187098.1  : ........................A....................................................................A...... : 300
KT187198.2  : ........................A....................................................................A...... : 300
KT186898.1  : ........................A....................................................................A...... : 300
KT715642.1  : ........................A....................................................................A...... : 300
KT186918.1  : ........................A....................................................................A...... : 300
KT187078.1  : ........................A....................................................................A...... : 300
KT186988.1  : ........................A....................................................................A...... : 300
KX987169.1  : ........................A....................................................................A...... : 300
KT186998.1  : ........................A....................................................................A...... : 300
KT186948.1  : ........................A....................................................................A...... : 300
KT186928.1  : ........................A....................................................................A...... : 300
AY163329.1  : ........................A....................................................................A...... : 300
KX987179.1  : ........................A....................................................................A...... : 300
AM883165.1  : ........................A....................................................................A...... : 300
KY471475.1  : ........................A....................................................................A...... : 300
OM401814.1  : ........................A....................................................................A...... : 300
```

Figure S1, to be continued-4

```
                   *        320         *        340         *        360         *        380         *        400
PX069094   : INVLLEYCESLTTFVHRKKRKEGDDLTARNTFRQALIKSMPTMNLKNQMKMTRGWGNYTFFSYIDRLSRIYNMNIDPNGKLWTEHKQTVSEQLKKKQEEN : 400
KP939375.1 : ............................................F.....................F...............I................. : 400
OM289920.1 : ............................................F.....................F...............I................. : 400
KT030471.1 : ............................................F.....................F...............V................. : 400
KT030461.1 : ............................................F.....................F...............V................. : 400
KT030501.1 : ............................................F.....................F...............V................. : 400
KT030481.1 : ............................................F.....................F...............V................. : 400
KP009622.1 : ............................................F.....................F...............V................. : 400
MT461278.1 : ............................................F.....................F...............V................. : 400
KP939377.1 : ............................................F.....................F...............N................. : 400
KP009712.1 : ...........I................................F.....................F................................. : 400
KT030441.1 : ............................................F...........K.........F...............I................. : 400
KP939373.1 : ............................................F...........K.........F...............I................. : 400
KT187088.1 : ............................................F...........K.........F...............I................. : 400
FJ183365.1 : ............................................F...........K.........F...............I................. : 400
KY471474.1 : ............................................F...........K.........F...............I................. : 400
KT187068.1 : ............................................F...........K.........F...............I................. : 400
KT030491.1 : ............................................F...........K.........F...............I................. : 400
KT187228.1 : ............................................F...........K.........F...............I................. : 400
KP939374.1 : ............................................F...........K.........F...............I................. : 400
KT070448.1 : ............................................F...........K.........F...............I................. : 400
KT070458.1 : ............................................F...........K.........F...............I................. : 400
KT070508.1 : ............................................F...........K.........F...............I................. : 400
KT187108.1 : ............................................F...........K.........F...............I................. : 400
KT187098.1 : ............................................F...........K.........F...............I................. : 400
KT187198.2 : ............................................F...........K.........F...............I................. : 400
KT186898.1 : ............................................F...........K.........F...............I................. : 400
KT715642.1 : ............................................F...........K.........F...............I................. : 400
KT186918.1 : ............................................F...........K.........F...............I................. : 400
KT187078.1 : ............................................F...........K.........F...............I................. : 400
KT186988.1 : ............................................F.....................F...............I................. : 400
KX987169.1 : ..........................I.................F.....................F...............I................. : 400
KT186998.1 : ............................................F...............S.....F...............I................. : 400
KT186948.1 : ............................................F.....................F...............I................. : 400
KT186928.1 : ............................................F.....................F...............I................. : 400
AY163329.1 : ............................................F.....................F...............I................. : 400
KX987179.1 : ..............................A.............F.....................F...............I................. : 400
AM883165.1 : ............................................F.....................F...............I................. : 400
KY471475.1 : ............................................F.....................F...............I................. : 400
OM401814.1 : ............................................F.....................F...............I................. : 400
```

```
                      *       420         *       440         *       460         *       480         *       500
PX069094   : RAPLTVQIDGVHIRTDETYGTVDHWVEWVVDTIMLRETEKMIKDYRFKKLKREELISGMNKLEDGLRCIVYCLILTLYDYYEGDIEGFKKGTIASSIVET : 500
KP939375.1 : Q...................................................D......................A........................ : 500
OM289920.1 : Q...................................................D......................A........................ : 500
KT030471.1 : ....................................................D......................A........................ : 500
KT030461.1 : ....................................................D......................A........................ : 500
KT030501.1 : ....................................................D......................A........................ : 500
KT030481.1 : ....................................................D......................A........................ : 500
KP009622.1 : ....................................................D......................A........................ : 500
MT461278.1 : ....................................................D......................A........................ : 500
KP939377.1 : ....................................................D......................A........................ : 500
KP009712.1 : .................................................................................................... : 500
KT030441.1 : ...........................................................................A........................ : 500
KP939373.1 : ..............A............................................................A........................ : 500
KT187088.1 : ...........................................................................A........................ : 500
FJ183365.1 : ...........................................................................A........................ : 500
KY471474.1 : ...........................................................................A........................ : 500
KT187068.1 : ...........................................................................A........................ : 500
KT030491.1 : ...........................................................................A........................ : 500
KT187228.1 : ...........................................................................A........................ : 500
KP939374.1 : ...........................................................................A........................ : 500
KT070448.1 : ...........................................................................A........................ : 500
KT070458.1 : ...........................................................................A........................ : 500
KT070508.1 : ...........................................................................A........................ : 500
KT187108.1 : ...........................................................................A........................ : 500
KT187098.1 : ...........................................................................A........................ : 500
KT187198.2 : ...........................................................................A........................ : 500
KT186898.1 : ...........................................................................A........................ : 500
KT715642.1 : ...........................................................................A........................ : 500
KT186918.1 : ...........................................................................A........................ : 500
KT187078.1 : ...........................................................................A........................ : 500
KT186988.1 : ...........................................................................A........................ : 500
KX987169.1 : ...........................................................................A........................ : 500
KT186998.1 : ...........................................................................A........................ : 500
KT186948.1 : ...........................................................................A........................ : 500
KT186928.1 : ...........................................................................A........................ : 500
AY163329.1 : ...........................................................................A........................ : 500
KX987179.1 : ...........................................................................A........................ : 500
AM883165.1 : ...........................................................................A........................ : 500
KY471475.1 : ...........................................................................A........................ : 500
OM401814.1 : ...........................................................................A........................ : 500
```

Figure S1, to be continued-6

```
                      *         520         *         540         *         560         *         580         *         600
PX069094   : VSQMFPNFRSDIIDKFGISLKVRTEAEELFLPKNMKSSMNVGEERGYKYKFGWKDNEEKVMSNYGKILTESVEILFKKLLKGEKWKIIVDDPQTYFEDDL : 600
KP939375.1 : .................................................................E....................T............. : 600
OM289920.1 : .................................................................E................K...T............. : 600
KT030471.1 : .................................................................E.................................. : 600
KT030461.1 : .................................................................E.................................. : 600
KT030501.1 : .................................................................E.................................. : 600
KT030481.1 : .................................................................E.................................. : 600
KP009622.1 : .................................................................E.................................. : 600
MT461278.1 : .................................................................E....................T............. : 600
KP939377.1 : .....................................P.S..............E..........E.................................. : 600
KP009712.1 : .....................A...........................................E.................................. : 600
KT030441.1 : .........................................R.......................E.................................. : 600
KP939373.1 : .................................................................E.................................. : 600
KT187088.1 : .................................................................E.................................. : 600
FJ183365.1 : .................................................................E.................................. : 600
KY471474.1 : .................................................................E.................................. : 600
KT187068.1 : .................................................................E.................................. : 600
KT030491.1 : .................................................................E.................................. : 600
KT187228.1 : .................................................................E.................................. : 600
KP939374.1 : .................................................................E.................................. : 600
KT070448.1 : .................................................................E.................................. : 600
KT070458.1 : .................................................................E.................................. : 600
KT070508.1 : .................................................................E.................................. : 600
KT187108.1 : .................................................................E.................................. : 600
KT187098.1 : .................................................................E.................................. : 600
KT187198.2 : .................................................................E.................................. : 600
KT186898.1 : .................................................................E.................................. : 600
KT715642.1 : .................................................................E.................................. : 600
KT186918.1 : .................................................................E.................................. : 600
KT187078.1 : .................................................................E.................................. : 600
KT186988.1 : .................................................................E.................................. : 600
KX987169.1 : .................................................................E.................................. : 600
KT186998.1 : .................................................................E.................................. : 600
KT186948.1 : .................................................................E.................................. : 600
KT186928.1 : .................................................................E.................................. : 600
AY163329.1 : .................................................................E.................................. : 600
KX987179.1 : .................................................................E.................................. : 600
AM883165.1 : .................................................................E.................................. : 600
KY471475.1 : .................................................................E.................................. : 600
OM401814.1 : .................................................................E.................................. : 600
```

Figure S1, to be continued-7

```
                         *       620         *       640         *       660         *       680         *       700
PX069094    : FVDRANKIFSKGGQTVDQLISIKVNAQSNKVEGTTYFSKRFVSYWFRIEHFSITTAKKRTDIRDKRTEYNEFDFEDFKPACIGELGIHASTYIYQDLLVG : 700
KP939375.1  : .............................N...................................................................... : 700
OM289920.1  : .............................N...................................................................... : 700
KT030471.1  : .............................N..........................E........................................... : 700
KT030461.1  : .............................N..........................E........................................... : 700
KT030501.1  : .............................N..D................................................................... : 700
KT030481.1  : .............................N...................................................................... : 700
KP009622.1  : .............................N...................................................................... : 700
MT461278.1  : .............................N.............................I........................................ : 700
KP939377.1  : .............................N...................................................................... : 700
KP009712.1  : .............................N...................................................................... : 700
KT030441.1  : .............................N...................................................................... : 700
KP939373.1  : .............................N...................................................................... : 700
KT187088.1  : .............................N...................................................................... : 700
FJ183365.1  : .............................N...................................................................... : 700
KY471474.1  : .............................N...................................................................... : 700
KT187068.1  : .............................N...................................................................... : 700
KT030491.1  : .............................N...................................................................... : 700
KT187228.1  : .............................N...................................................................... : 700
KP939374.1  : .............................N...................................................................... : 700
KT070448.1  : .............................N...................................................................... : 700
KT070458.1  : .............................N...................................................................... : 700
KT070508.1  : .............................N...................................................................... : 700
KT187108.1  : .............................N...................................................................... : 700
KT187098.1  : .............................N...................................................................... : 700
KT187198.2  : .............................N...................................................................... : 700
KT186898.1  : .............................N...................................................................... : 700
KT715642.1  : .............................N...................................................................... : 700
KT186918.1  : .............................N...................................................................... : 700
KT187078.1  : .............................N...................................................................... : 700
KT186988.1  : .............................N...................................................................... : 700
KX987169.1  : .............................N...................................................................... : 700
KT186998.1  : .............................N...................................................................... : 700
KT186948.1  : .............................N...................................................................... : 700
KT186928.1  : .............................N...................................................................... : 700
AY163329.1  : ...............A.............N...................................................................... : 700
KX987179.1  : .............................N...................................................................... : 700
AM883165.1  : .............................N...................................................................... : 700
KY471475.1  : .............................N...................................................................... : 700
OM401814.1  : .............................N...................................................................... : 700
```

```
                        *       720         *       740         *       760         *       780         *       800
PX069094    : RSRGERVKDAKELVWMDLSLANFGCSRCYDRCWPASCVEAEISLRYHLVTSIFTRYLNREGLSFSKILNSLKDFSDRLWFPTYKHFYVAVVQKVLRDDRR : 800
KP939375.1  : ..............................................Y......A..............I............................... : 800
OM289920.1  : ..............................................Y......A..............I............................... : 800
KT030471.1  : ..............................................Y......A..............I............................... : 800
KT030461.1  : ..............................................Y......A..............I............................... : 800
KT030501.1  : ..............................................Y......A..............I............................... : 800
KT030481.1  : ..............................................Y......A..............I............................... : 800
KP009622.1  : ..............................................Y......A..............I............................... : 800
MT461278.1  : ..............................................Y......A..............I............................... : 800
KP939377.1  : ..............................................Y......A..............I............................... : 800
KP009712.1  : .................................................................................................... : 800
KT030441.1  : ..............................................Y......A..............I............................... : 800
KP939373.1  : ..............................................Y......A..............I............................... : 800
KT187088.1  : ..............................................Y......A..............I............................... : 800
FJ183365.1  : ..............................................Y......A..............I............................... : 800
KY471474.1  : ..............................................Y......A..............I............................... : 800
KT187068.1  : ..............................................Y......A..............I............................... : 800
KT030491.1  : ..............................................Y......A..............I............................... : 800
KT187228.1  : ..............................................Y......A..............I............................... : 800
KP939374.1  : ..............................................Y......A..............I............................... : 800
KT070448.1  : ..............................................Y......A..............I............................... : 800
KT070458.1  : ..............................................Y......A..............I............................... : 800
KT070508.1  : ..............................................Y......A..............I............................... : 800
KT187108.1  : ..............................................Y......A..............I............................... : 800
KT187098.1  : ..............................................Y......A..............I............................... : 800
KT187198.2  : ..............................................Y......A..............I............................... : 800
KT186898.1  : ..............................................Y......A..............I............................... : 800
KT715642.1  : ..............................................Y......A..............I............................... : 800
KT186918.1  : ..............................................Y......A..............I............................... : 800
KT187078.1  : ..............................................Y......A..............I............................... : 800
KT186988.1  : ..............................................Y......A..............I............................... : 800
KX987169.1  : ..............................................Y......A..............I............................... : 800
KT186998.1  : ..............................................Y......A..............I............................... : 800
KT186948.1  : ..............................................Y......A..............I............................... : 800
KT186928.1  : ..............................................Y......A..............I............................... : 800
AY163329.1  : ..............................................Y......A..............I............................... : 800
KX987179.1  : ..............................................Y......A..............I............................... : 800
AM883165.1  : ..............................................Y......A..............I............................... : 800
KY471475.1  : ..............................................Y......A..............I............................... : 800
OM401814.1  : ..............................................Y......A..............I............................... : 800
```

```
                      *       820         *       840         *       860         *       880         *       900
PX069094   : LDYVLFCSRISAITTRRAALMEFSTFKQMVESTRLLDTLFLNFLLWIIFEQENIDVDFANKWHPLLISAKKGLRVIAVDVFNSSLTLSMSGWLPYLERIC :  900
KP939375.1 : ..................T...........G.....................................TE.............................. :  900
OM289920.1 : ..................T...........G.....................................TE.............................. :  900
KT030471.1 : ..N...............T...........G.....................................TE.............................. :  900
KT030461.1 : ..N...............T...........G.....................................TE.............................. :  900
KT030501.1 : ..N...............T...........G.....................................TE.............................. :  900
KT030481.1 : ..N...............T...........G.....................................TE.............................. :  900
KP009622.1 : ..N...............T...........G.....................................TE.............................. :  900
MT461278.1 : ..N...............T...........G.....................................TE.............................. :  900
KP939377.1 : ..................T...........G.....................................TE.............................. :  900
KP009712.1 : ....................................................................TE............G................. :  900
KT030441.1 : ..................T...........G.....................................TE..................T........... :  900
KP939373.1 : ..................T...........G.....................................TE..................T........... :  900
KT187088.1 : ..................T...........G.....................................TE..................T........... :  900
FJ183365.1 : ..................T...........G.....................................TE..................T........... :  900
KY471474.1 : ..................T...........G.....................................TE..................T........... :  900
KT187068.1 : ..................T...........G.....................................TE..................T........... :  900
KT030491.1 : ..................T...........G.....................................TE..................T........... :  900
KT187228.1 : ..................T...........G.....................................TE..................T........... :  900
KP939374.1 : ..................T...........G.....................................TE..................T........... :  900
KT070448.1 : ..................T...........G.....................................TE..................T........... :  900
KT070458.1 : ..................T...........G.....................................TE..................T........... :  900
KT070508.1 : ..................T...........G.....................................TE.............................. :  900
KT187108.1 : ..................T...........G.....................................TE..................T........... :  900
KT187098.1 : ..................T...........G.....................................TE..................T........... :  900
KT187198.2 : ..................T...........G.....................................TE..................T........... :  900
KT186898.1 : ..................T...........G.....................................TE..................T........... :  900
KT715642.1 : ..................T...........G.....................................TE..................T........... :  900
KT186918.1 : ..................T...........G.....................................TE..................T........... :  900
KT187078.1 : ..................T...........G.....................................TE..................T........... :  900
KT186988.1 : ..................T...........G.....................................TE..........L.......T........... :  900
KX987169.1 : ..................T...........G.....................................TE..................T........... :  900
KT186998.1 : ..................T...........G.....................................TE..................T........... :  900
KT186948.1 : ..................T...........G.....................................TE..................T........... :  900
KT186928.1 : ..................T...........G.....................................TE..................T........... :  900
AY163329.1 : ..................T...........G.....................................TE..................T........... :  900
KX987179.1 : ..................T...........G.....................................TE..................T........... :  900
AM883165.1 : ..................T...........G.....................................TE..................T........... :  900
KY471475.1 : ..................T...........G.....................................TE..................T........... :  900
OM401814.1 : ..................T...........G.....................................TE..................T........... :  900
```

Figure S1, to be continued-10

```
                      *         920         *         940         *         960         *         980         *        1000
PX069094   : SESVMDRALTVDEINLKRWFVDYYMELKLERRAEPRMSFKSEALITWIGSNCGGVTDYVVQLLPVRKPKPGLLVVVYSEDGGEKWAEWALRDYLEIDGSL : 1000
KP939375.1 : ...A......A......................................................................................... : 1000
OM289920.1 : ...A......A......................................................................................... : 1000
KT030471.1 : ...A.....AA......................................................................................... : 1000
KT030461.1 : ...A.....AA......................................................................................... : 1000
KT030501.1 : ...A.....AA......................................................................................... : 1000
KT030481.1 : ...A.....AA......................................................................................... : 1000
KP009622.1 : ...A.....AA......................................................................................... : 1000
MT461278.1 : ...A.....AA......................................................................................... : 1000
KP939377.1 : ...A......A......................................................................................... : 1000
KP009712.1 : ..........A......................................................................................... : 1000
KT030441.1 : ...A......A......................................................................................... : 1000
KP939373.1 : ...A......A......................................................................................... : 1000
KT187088.1 : ...A......A......................................................................................... : 1000
FJ183365.1 : ...A......A......................................................................................... : 1000
KY471474.1 : ...A......A......................................................................................... : 1000
KT187068.1 : ...A......A......................................................................................... : 1000
KT030491.1 : ...A......A......................................................................................... : 1000
KT187228.1 : ...A......A......................................................................................... : 1000
KP939374.1 : ...A......A......................................................................................... : 1000
KT070448.1 : ...A......A......................................................................................... : 1000
KT070458.1 : ...A......A......................................................................................... : 1000
KT070508.1 : ...A......A......................................................................................... : 1000
KT187108.1 : ...A......A......................................................................................... : 1000
KT187098.1 : ...A......A......................................................................................... : 1000
KT187198.2 : ...A......A......................................................................................... : 1000
KT186898.1 : ...A......A......................................................................................... : 1000
KT715642.1 : ...A......A......................................................................................... : 1000
KT186918.1 : ...A......A......................................................................................... : 1000
KT187078.1 : ...A......A......................................................................................... : 1000
KT186988.1 : ...A......A......................................................................................... : 1000
KX987169.1 : ...A......A......................................................................................... : 1000
KT186998.1 : ...A......A......................................................................................... : 1000
KT186948.1 : ...A......A......................................................................................... : 1000
KT186928.1 : ...A......A......................................................................................... : 1000
AY163329.1 : ...A......A......................................................................................... : 1000
KX987179.1 : ...A......A......................................................................................... : 1000
AM883165.1 : ...A......A......................................................................................... : 1000
KY471475.1 : ...A......A......................................................................................... : 1000
OM401814.1 : ...A......A......................................................................................... : 1000
```

```
                      *      1020         *      1040         *
PX069094    : GLVFITRKAVKNKSKLGVRDLKIYNRGRVDRLILISSGVYTFGNKFLFSKLLSKIE : 1056
KP939375.1  : ........................................................ : 1056
OM289920.1  : ........................................................ : 1056
KT030471.1  : ........................................................ : 1056
KT030461.1  : ........................................................ : 1056
KT030501.1  : ........................................................ : 1056
KT030481.1  : ........................................................ : 1056
KP009622.1  : ........................................................ : 1056
MT461278.1  : ........................................................ : 1056
KP939377.1  : ........................................................ : 1056
KP009712.1  : ........................................................ : 1056
KT030441.1  : ........................................................ : 1056
KP939373.1  : ........................................................ : 1056
KT187088.1  : ........................................................ : 1056
FJ183365.1  : ........................................................ : 1056
KY471474.1  : ........................................................ : 1056
KT187068.1  : ........................................................ : 1056
KT030491.1  : ........................................................ : 1056
KT187228.1  : ........................................................ : 1056
KP939374.1  : ........................................................ : 1056
KT070448.1  : ........................................................ : 1056
KT070458.1  : ........................................................ : 1056
KT070508.1  : ........................................................ : 1056
KT187108.1  : ........S............................................... : 1056
KT187098.1  : ........S............................................... : 1056
KT187198.2  : ........S............................................... : 1056
KT186898.1  : ........................................................ : 1056
KT715642.1  : ........................................................ : 1056
KT186918.1  : ........................................................ : 1056
KT187078.1  : ........................................................ : 1056
KT186988.1  : ........................................................ : 1056
KX987169.1  : ........................................................ : 1056
KT186998.1  : ........................................................ : 1056
KT186948.1  : ........................................................ : 1056
KT186928.1  : ........................................................ : 1056
AY163329.1  : ........................................................ : 1056
KX987179.1  : ........................................................ : 1056
AM883165.1  : ........................................................ : 1056
KY471475.1  : ........................................................ : 1056
OM401814.1  : ........................................................ : 1056
```

Figure S1. Comparison of the deduced amino acid sequences of AHSV-1 VP2. 39 deduced amino acid sequences of AHSV-1 VP2 downloaded from GenBank were aligned with the deduced amino acid sequence of AHSV/C VP2 (accession no.: PX069094), which served as the reference sequence. Amino acids identical to those in the reference sequence are denoted by dots, whereas amino acids differing from the reference sequence are indicated by their corresponding one-letter symbols. Underlined regions and the rectangular box mark the positions of linear epitopes recognized by the two mouse monoclonal antibodies, respectively.
